# In Vitro Anti-Inflammatory and Radical Scavenging Properties of Chinotto (*Citrus myrtifolia* Raf.) Essential Oils

**DOI:** 10.3390/nu10060783

**Published:** 2018-06-18

**Authors:** Pierluigi Plastina, Astari Apriantini, Jocelijn Meijerink, Renger Witkamp, Bartolo Gabriele, Alessia Fazio

**Affiliations:** 1 Department of Pharmacy, Health and Nutritional Sciences, University of Calabria, 87036 Arcavacata di Rende (CS), Italy; astariapriantini@yahoo.com; 2 Division of Human Nutrition, Wageningen University, 6700 AA Wageningen, The Netherlands; jocelijn.meijerink@wur.nl (J.M.); renger.witkamp@wur.nl (R.W.); 3 Department of Chemistry and Chemical Technologies, University of Calabria, 87036 Arcavacata di Rende (CS), Italy; bartolo.gabriele@unical.it

**Keywords:** antioxidant, *Citrus*, inflammation, macrophages, nitric oxide

## Abstract

Chinotto (*Citrus myrtifolia* Raf.) is a widely diffused plant native from China and its fruits have a wide-spread use in confectionary and drinks. Remarkably, only little has been reported thus far on its bioactive properties, in contrast to those of the taxonomically related bergamot (*Citrus bergamia* Risso). The present study aimed to investigate potential in vitro anti-inflammatory and radical scavenging properties of chinotto essential oils (CEOs) and to establish to what extent their composition and bioactivities are dependent on maturation. Essential oil from half ripe chinotto (CEO2) reduced the production of nitric oxide (NO) and the expression of inflammatory genes, cyclooxygenase-2 (COX-2) and inducible nitric oxide synthase (iNOS), cytokines, including interleukin-1β (IL-1β) and interleukin-6 (IL-6), and chemokine monocyte chemotactic protein-1 (MCP-1) by lipopolysaccharide (LPS)-stimulated RAW264,7 macrophages. Limonene, linalool, linalyl acetate, and γ-terpinene were found to be the main components in CEO2. Moreover, CEO2 showed high radical scavenging activity measured as Trolox equivalents (TE) against both 2,2′-diphenyl-1-picrylhydrazyl (DPPH) and 2,2′-azino-bis(3-ethylbenzothiazoline-6-sulfonic acid) diammonium salt (ABTS). These findings show that chinotto essential oil represents a valuable part of this fruit and warrants further *in vivo* studies to validate its anti-inflammatory potential.

## 1. Introduction

Inflammation and oxidative stress are essential to maintain homeostasis and provide, among others, protection to pathogens or tissue damage. However, under certain conditions, these mechanisms are also involved in chronic low-grade inflammatory processes, which, even in the absence of immediate clinical symptoms, present risk factors for many diseases [1,2,3,4,5]. Macrophages are key players in the early stages of an inflammatory response, which can either stay controlled and, eventually, resolve or develop into chronic inflammation [6,7]. The second situation is characterized by the release of pro-inflammatory mediators, including nitric oxide (NO) and cyclooxygenase-2 (COX-2) derived eicosanoids, as well as cytokines and chemokines, including interleukin (IL)-1β, IL-6, and tumor necrosis factor (TNF)-α. Pro-inflammatory macrophages play important roles in these situations, contributing to various co-morbidities [8,9].

Consumption of vegetables, fruit, and herbs is associated with positive health effects, not only because of their favourable nutrient composition, but also because of the presence of different secondary metabolites, several of which have been shown to contribute to a reduced inflammatory tone [10,11]. Essential oils represent an important group in this respect, which is also reflected in their widespread traditional use in inflammatory disorders [12,13]. More recently, essential oils have also become of interest as natural additives or ingredients of functional foods, with claimed beneficial health properties [14,15].

*Citrus myrtifolia* Raf., commonly known as chinotto or myrtle leaved orange, is a widely diffused ornamental plant belonging to the *Rutaceae* family and is considered as a mutation of sour orange (*Citrus aurantium* L.) [16,17]. The plant is native to China and has been cultivated for centuries in France and Italy, especially in Liguria, Calabria, and Sicily where the fruits are used in the confectionery industry. Next to this, the juice from unripe fruits is used as an ingredient of soft drinks and liqueurs [18]. Despite its many applications and its taxonomically close relationship to bergamot (*Citrus bergamia* Risso) [19,20], whose beneficial effects on inflammation and inflammatory-related disorders are being recognized [21,22,23,24,25,26,27], there appear to be no documented studies on the potential anti-inflammatory properties of chinotto. Moreover, although the composition of different parts of the chinotto plant has been reported [18,28,29,30,31], including that of the essential oil of the peels [32,33], the effect of fruit maturation on the essential oil profile and on potential differences in bioactivity seems to be neglected thus far. These starting points prompted us to investigate the in vitro anti-inflammatory and radical scavenging potential of essential oils from the peels of chinotto and to evaluate the effects of maturation on the bioactivity and on the composition of volatile compounds.

## 2. Materials and Methods

### 2.1. Chemicals

*n*-Hexane, chloroform, and methanol (analytical grade) were purchased from Carlo Erba Reagenti (Milan, Italy). β-Pinene, limonene, sabinene, myrcene, γ-terpinene, linalool, neral, linalyl acetate, geranial, geranyl acetate, and β-caryophyllene were supplied by Fluka (Milan, Italy). 2,2′-Diphenyl-1-picrylhydrazyl (DPPH) and 2,2′-azino-bis(3-ethylbenzothiazoline-6-sulfonic acid) diammonium salt (ABTS), and (±)-6-hydroxy-2,5,7,8-tetramethylchromane-2-carboxylic acid (Trolox) were from Sigma-Aldrich (Milan, Italy). Dulbecco’s modified Eagle’s medium (DMEM), fetal bovine serum, streptomycin, and penicillin were acquired from Lonza (Verviers, Belgium). Lipopolysaccharides (LPS, *E. coli* O111:B4) was obtained from Sigma-Aldrich (Schnelldorf, Germany). Griess reagents and nitrite standard were purchased from Cayman Chemical (Ann Arbor, MI, USA).

### 2.2. Plant Material and Extraction of the Oils

Chinotto fruits were collected in Calabria (southern Italy) in three different periods (early October, late October, late November) during Autumn 2012. A voucher specimen (accession no. CLU26013) of the plant was deposited in the Erbarium CLA at the Botanical Garden of the University of Calabria (Rende, Italy). Chinotto essential oils (CEOs) were obtained according to a previously reported extraction method [34]. Briefly, the rinds of the peels were squeezed to break the utricles and release the oil, which was collected by extraction with *n*-hexane. The solutions were then dried over Na_2_SO_4_ and concentrated under a stream of N_2_. Extracts were classified as CEO1, CEO2, or CEO3 based on the ripening stage of the fruits used (green, half ripe, and ripe, respectively). Specifically, unripe fruits were green and half ripe were yellow-green, whereas ripe fruits were fully orange. The diameter was about 4–6 cm in all the cases. The essential oils were stored under N_2_ at −20 °C in brown bottles until analyses.

### 2.3. GC Analyses of CEOs

The composition of CEOs was analysed by gas chromatography using a flame ionization detector (GC-FID) without any derivatisation. Before GC analysis, CEOS were diluted 10 mg in 1 mL chloroform. Analyses were carried out on a Shimadzu GC-2010 system equipped with an AOC-20i auto sampler, split/split less injector, and a FID detector (Shimadzu, Milan, Italy). Experimental conditions were as follows: The column was a fused-silica capillary column (SLB^™^-5 ms, Supelco, Milan, Italy) coated with 5% diphenyl −95% dimethyl siloxane (30 m × 0.25 mm id × 0.25 μm d_f_); the oven temperature increased from 50 up to 250 °C, with a rate of 3.0 °C min^−1^; and the injection volume was 1.0 μL in the split mode (30:1). Helium was used as carrier gas at 30.1 cm s^−1^ of linear velocity (*u*), with an inlet pressure of 99.8 kPa. The detector temperature was set at 280 °C. The hydrogen flow rate was 50.0 mL min^−1^; the air flow rate was 400 mL min^−1^; and the make-up flow rate (N_2_/Air) was 50 mL min^−1^. The identification of the compounds was based on the comparison of their retention times with those of authentic standards available in our laboratory. The quantification of each compound identified in CEOs was performed using the peak area normalization and the results were expressed as the means of three experiments. 

### 2.4. In Vitro Radical Scavenging Activity

#### 2.4.1. DPPH Assay

The free radical scavenging capacities of the oils were determined by DPPH assay according to a previously used protocol [35]. In particular, 100 μL of CEOs (10 mg mL^−1^ MeOH) were mixed with 100 μL of the DPPH• methanol solution (1 mM) and the final volume adjusted to 3 mL by the addition of the necessary amount of MeOH. Next, the mixtures were shaken vigorously and incubated in the dark at room temperature. The colorimetric decrease in absorbance of each sample was measured at 517 nm using a UV–Vis spectrophotometer (model V-550, Jasco Europe) after 30 min when a plateau was reached. The negative control was a DPPH solution obtained by diluting 100 μL of the DPPH standard solution with MeOH to give a final volume of 3 mL. Experiments were carried out in triplicate. Trolox, a known antioxidant, has been used as reference compound to build a calibration curve and the results are expressed as µmol of Trolox Equivalents (TE)/g of CEO. 

#### 2.4.2. ABTS Assay

The ABTS assay was conducted according to an established protocol [36], with slight modifications. ABTS radical cation (ABTS•^+^) was produced by reacting ABTS solution (7 mM) with potassium persulfate (2.45 mM) and allowing the mixture to stand in the dark at room temperature for 16 h before use. The ABTS•^+^ solution was diluted with ethanol and the resulting solution had an absorbance of 0.70 ± 0.05 (at 734 nm). 100 μL of the CEOs (10 mg mL^−1^) were added to 2 mL of ABTS•^+^ solution and the resulting mixture was kept in the dark and under stirring for 5 min before absorbance at 734 nm was measured. Experiments were carried out in triplicate. Trolox was used as the reference compound to build a calibration curve and the results are expressed as µmol of Trolox Equivalents (TE)/g of CEO. 

### 2.5. Cell Culture

RAW264.7 cells, a mouse-derived macrophage cell line, were obtained from the American Type Culture Collection (Teddington, UK). The cells were grown in Dulbecco’s modified Eagle’s medium (DMEM) containing 10% fetal bovine serum, streptomycin, and penicillin at 37 °C in a 5% CO_2_ humidified air atmosphere. Cells were seeded into 96-well cell culture plates (2.5 × 10^5^ cells mL^−1^) for NO and viability analyses or in 6-well plates (5 × 10^5^ cells mL^−1^) for RNA extraction, and incubated overnight. Adherent cells were incubated with LPS (0.5 mg mL^−1^) with (or without) the test CEOs for 48 h (for nitrite and viability measurements) or 24 h (for RNA extraction).

### 2.6. XTT Viability Assay

Effects of CEOs on cell viability were analysed using an XTT Cell Proliferation Kit II (Roche Applied Science, Almere, The Netherlands) following the manufacturer’s instructions. After 48 h, the tetrazolium salt (MTT) assay was performed, evaluating the cells’ capability in metabolising XTT to formazan as an estimation of cell viability. Conditions were considered toxic if metabolic activity leading to formazan was diminished by ≥20%. As a positive control, cells were treated with Triton X100, yielding total cell lysis. 

### 2.7. Nitric Oxide Quantification 

Nitrite present in the culture medium, after incubation for 48 h, was measured using Griess reagents to give an estimation of NO production [37]. Briefly, 100 μL of the cell supernatant were reacted with 100 μL of Griess reagents and incubated at room temperature for 10 min. Absorbance was determined at 540 nm using an ELISA plate reader. 

### 2.8. RNA Extraction, Purification, and Quantitative Reverse Transcription Real-Time PCR

After incubating for 24 h, medium was removed and total RNA was extracted using TRIzol (Invitrogen, Breda, The Netherlands). RNA (1 μg/sample) was reversely transcribed, yielding cDNA using Promega reverse transcription system (Leiden, The Netherlands). cDNA was amplified by PCR by means of platinum Taq DNA polymerase (Invitrogen) and SYBR green (Molecular Probes, Leiden, The Netherlands) using an iCycler system (Bio-Rad, Veenendaal, The Netherlands). The following primer pairs were used for amplification of iNOS: 5′-GTTCTCAGCCCAACAATACAAGA-3′ (forward) e 5′-GTGGACGGGTCGATGTCAC-3′ (reverse); COX-2: 5′-GGAGAGACTATCAAGATAGT-3′ (forward) and 5′-ATGGTCAGTAGACTTTTACA-3′ (reverse); IL-1β: 5′-TGCAGAGTTCCCCAACTGGTACATC-3′ (forward) and 5′-GTGCTGCCTAATGTCCCCTTGAATC-3′ (reverse). IL-6: 5′-TACTCGGCAAACCTAGTGCG-3′ (forward) and 5′-GTGTCCCAACATTCATATTGTCAGT-3′ (reverse); MCP-1: 5′-CCCAATGAGTAGGCTGGAGA-3′ (forward) and 5′-TCTGGACCCATTCCTTCTTG-3′ (reverse). Samples were analysed in duplicate, and mRNA expression levels of the different genes were normalised to RPS27A2. Primer pairs for RPS27A2 were 5′-GGTTGAACCCTCGGACACTA-3′ (forward) and 5′-GCCATCTTCCAGCTGCTTAC-3′ (reverse).

### 2.9. Statistical Analysis

All experiments were performed in duplicate in at least three independent experiments. Data from all experiments using RAW264.7 macrophages are presented as a percentage of the LPS-treated controls (set at 100%). Data from radical scavenging assays are expressed as µmol of Trolox Equivalents (TE)/g of CEO. All data are reported as means ± standard deviation. Statistical differences between treatments were evaluated by one-way ANOVA followed by Bonferroni’s post hoc test. *p* values < 0.05 (*) and <0.01 (**) were considered as statistically significant. 

## 3. Results 

### 3.1. Composition of CEOs

The quantitative recovery of CEOs was 0.7% (*w*/*w*; based on fresh dried peels) for CEO1 and CEO2 and 0.4% (*w*/*w*) for CEO3. The composition of CEOs was analysed by GC and the results are reported in Table 1. Twelve compounds were identified and quantified in green, half ripe, and ripe fruit essential oils, representing 96.7%, 95.9%, and 97.5% of the total detected constituents, respectively. Limonene, linalool, and linalyl acetate were identified as the main components in all the oils, while γ-terpinene was found in CEO2 and CEO3 (3.7% and 0.4%, respectively). The sum of these four monoterpenes accounted for 94.0%, 93.0%, and 93.5% of the total constituents in the three CEOs, respectively. In particular, limonene was found to be the most abundant compound in CEO2 and CEO3 (54.3 and 48.7%, respectively). Linalool content reached its highest concentration in CEO3 (32.4%), while linalyl acetate was found to be the most abundant compound in CEO1 (47.5%) and its amount decreased significantly upon ripening. 

### 3.2. In Vitro Radical Scavenging Properties of CEOs

The radical scavenging activity of CEOs was evaluated against the two most commonly used stable radicals (DPPH• and ABTS•^+^). As can be seen in Table 2, DPPH• scavenging activity of CEOs increased with ripening. In particular, CEO2 and CEO3 (7.8 and 8.1 µmol of TE/g of CEO, respectively) were significantly more active than CEO1 (6.1 µmol of TE/g of CEO). An opposite trend was observed for the ABTS assay, where CEO1 and CEO2 had higher scavenging activity (11.1 and 10.8 µmol of TE/g of CEO, respectively) than CEO3 (9.4 µmol of TE/g of CEO).

### 3.3. CEOs Have No Effect on Cell Proliferation

**Figure 1:**
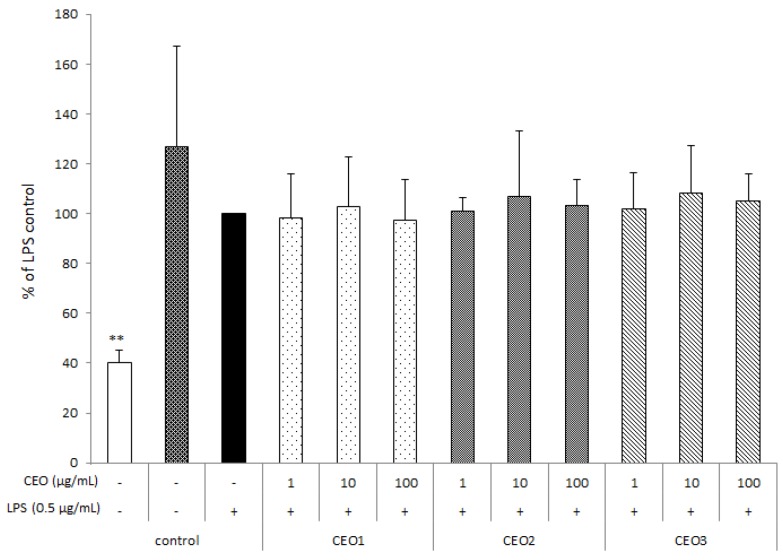
Effect of chinotto essential oils at different ripening stages [CEO1 (green), CEO2 (half ripe), and CEO3 (ripe)] on the cell viability of RAW264.7 macrophages. Cells were seeded in 96-well plates at a density of 2.5 × 10^5^ cells mL^−1^ and, after overnight incubation, were treated with lipopolysaccharide (LPS, 0.5 μg mL^−1^) and with (or without) increasing concentrations of CEOs for 48 h. Data are presented as percentages and LPS control (without CEOs) was fixed at 100% (black bar). The white bar represents the positive control, corresponding to untreated cells upon addition of Triton X100. The means of three separate experiments (each done in duplicate) ± standard deviation are reported. Mean value was statistically different from control (** *p* < 0.01).

Prior to nitrite determination, effects on cell proliferation by means of XTT analysis of the CEOs were evaluated at concentrations of 1, 10, and 100 µg mL^−1^. Figure 1 shows that CEOs had no significant effects on cell viability, with respect to LPS control (black bar). The white bar represents the positive control, corresponding to CEO unexposed cells upon addition of Triton X100, leading to total cell lysis.

### 3.4. CEO2 Reduces No Concentration in LPS-Stimulated Macrophages

**Figure 2:**
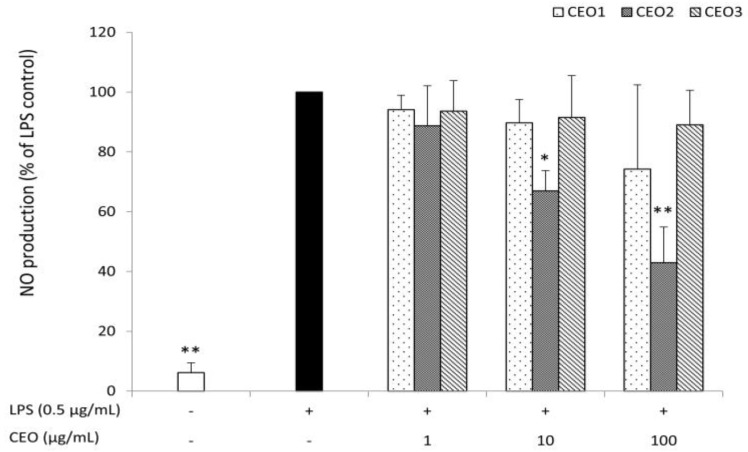
Effect of chinotto essential oils at different ripening stages [CEO1 (green), CEO2 (half ripe), and CEO3 (ripe)] on nitric oxide (NO) production by lipopolysaccharide (LPS)-stimulated RAW264.7 macrophages. Cells were seeded in 96-well plates at a density of 2.5 × 10^5^ cells mL^−1^ and, after overnight incubation, were treated with LPS (0.5 μg mL^−1^) and with (or without) increasing concentrations of CEOs for 48h. The supernatants of the cells were analysed for nitrite production by the Griess method. Data are presented as percentages and LPS control (without CEOs) was fixed at 100%. The average absolute nitrite value for the LPS control was approximately 40 μM. Values represent the mean of three independent experiments (each done in duplicate) ± standard deviation. Mean value was statistically different from control (* *p* < 0.05, and ** *p* < 0.01).

Chinotto essential oils were analysed for their capacity to reduce NO production by RAW264,7 macrophages stimulated by 0.5 µg mL^−1^ lipopolysaccharide (LPS). NO concentrations measured as nitrite were strongly induced after stimulation with LPS for 48 h. Essential oil from half ripe fruits (CEO2) reduced the level of NO in a concentration-dependent way. In particular, CEO2 was found to be already effective at 10 µg mL^−1^, while an inhibition of >50% was found at 100 µg mL^−1^. In contrast, CEO1 and CEO3 were ineffective in the concentration range investigated (Figure 2). 

### 3.5. CEO2 Attenuates LPS-Induced COX-2, iNOS, IL-1β, IL6, and MCP-1 Expression

**Figure 3:**
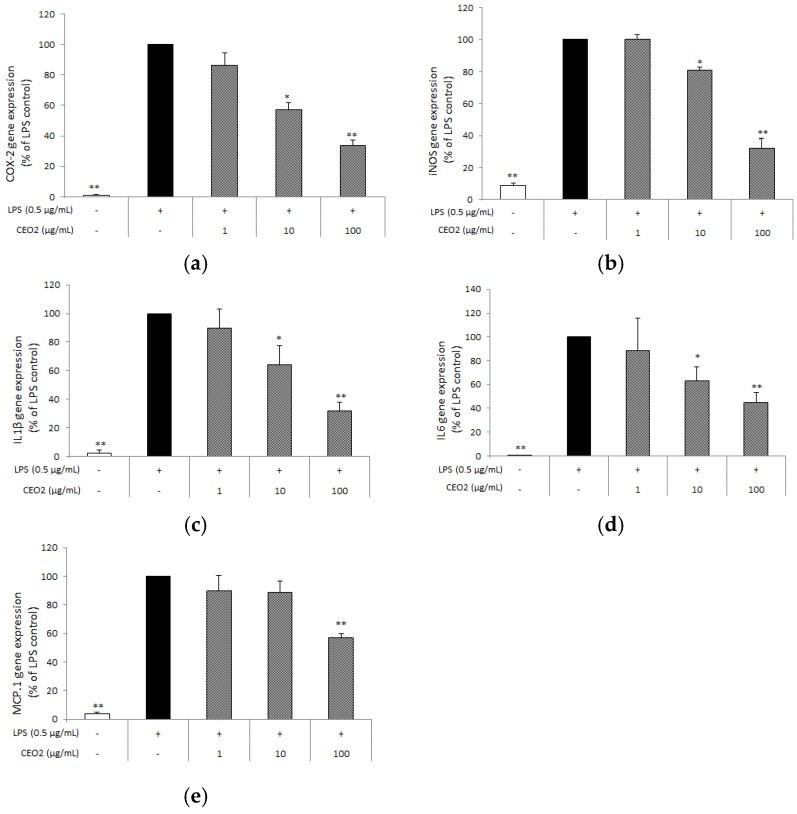
Effect of CEO2 on the lipopolysaccharide (LPS)-induced COX2 (**a**), iNOS (**b**), IL-1β (**c**), IL-6 (**d**), MCP-1 (**e**) gene expression in RAW264.7 macrophages. Cells were seeded in 6-well plates at a density of 5 × 10^5^ cells mL^−1^ and, after overnight incubation, were treated for 24 h with LPS (0.5 μg mL^−1^) and with (or without) different concentrations of CEO2. Total RNA was isolated and reverse transcribed, yielding cDNA prior to quantitative real-time PCR. Gene expression fold increase normalized to RPS27A2 is presented as percentages, and LPS stimulation (without CEO2) was fixed at 100%, and represents the mean of three separate experiments (each done in duplicate) ± standard deviation. Mean value was statistically different from control (* *p* < 0.05, and ** *p* < 0.01).

Subsequently, we investigated whether CEO2 also affected other key inflammatory mediators. Using quantitative RT-PCR, we established that CEO2 decreased cyclooxygenase-2 (COX-2) gene expression in a concentration-dependent way, with a reduction of 43% at 10 µg mL^−1^ and 64% at 100 µg mL^−1^ (Figure 3a). Similarly, CEO2 inhibited the gene expression of the cytokines, IL-1β and IL-6, as well as of iNOS, in a concentration-dependent way (Figure 3b–d). In particular, our results show that CEO2 decreased the expression of these genes by 36%, 37%, and 19% at 10 µg mL^−1^ and 68%, 55%, and 68% at 100 µg mL^−1^, respectively. The chemokine monocyte chemotactic protein-1 (MCP-1) was also reduced of 43% by CEO2 at the highest concentration (Figure 3e). 

## 4. Discussion

Nitric oxide (NO) is the free radical product of the oxidative deamination of l-arginine, which is catalysed by nitric oxide synthases (NOS). At low concentrations, NO plays a role as a signalling molecule in various physiological processes. During inflammation, the inducible isoform of the enzyme (iNOS or NOS2) is up-regulated and this produces large amounts of NO, acting as a key mediator in several inflammatory disorders [38,39]. Under these conditions, the expression of iNOS is known to be predominantly regulated at the transcriptional level [37]. Our results showed that the essential oil from half ripe fruits (CEO2) of chinotto (*Citrus myrtifolia* Raf.) could reduce NO levels in a concentration-dependent way. Moreover, CEO2 elicited a suppression of iNOS mRNA, indicating that the inhibition of NO release is, at least partly, mediated at a transcriptional level. Additionally, we found that CEO2 was also able to suppress the expression of other pro-inflammatory genes, including those encoding for cyclooxygenase-2 (COX-2), cytokines, interleukin-1β (IL-1β) and interleukin-6 (IL-6), and chemokine monocyte chemotactic protein-1 (MCP-1). It has been reported by us and others that the inhibition of COX-2-mediated eicosanoid production plays a major role in the reduction of pro-inflammatory mediators, including NO [37,40,41]. The upregulated COX-2 enzyme catalyses the reaction of arachidonic acid yielding prostaglandins, which function in inflammatory and pro-analgesic processes. Because of its pivotal role, COX-2 represents an important therapeutic target in many inflammatory disorders [37,40]. However, many COX-2 inhibitors (nonsteroidal anti-inflammatory drugs, NSAIDs) cause gastrointestinal side effects, and plant-derived alternatives targeting COX-2 are currently considered a promising strategy [42,43]. The pro-inflammatory mediators, IL-1β and IL-6, are expressed at high levels under inflammatory conditions [44]. In particular, IL-1β is an inducible cytokine that is, generally, not produced by healthy cells and it is implicated in pain, inflammation, and autoimmune conditions. Results from *in vivo* studies using IL-1β knock-out mice highlight the crucial role of IL-1β in mediating NF-κB activity and COX-2 transcription in response to systemic inflammation [45,46]. MCP-1 plays an important role in inducing macrophage infiltration to the site of inflammation, thus, leading to amplification of the inflammatory response [47]. In line with our findings, inhibitory effects of some essential oils, including those from thyme (*Thymus vulgaris*), oregano (*Origanum vulgare*), artemisia (*Artemisia fukudo*), and vetiver grass (*Vetiveria zizanioides*), have been reported to influence the production of the pro-inflammatory mediators, NO, prostaglandin E_2_ (PGE_2_), IL-1β, and IL-6, which was found to be mainly regulated at the transcriptional level [14,48,49,50]. Collectively, our results demonstrate, for the first time, that essential oils from chinotto possess anti-inflammatory properties similarly to other valuable *Citrus* fruits [12,13,51,52,53,54]. In particular, essential oil (EO) from sour orange (*Citrus aurantium* L.) has been recently reported to inhibit NO, IL-6, TNF-α, and IL-1β production, as well as their gene expression level in LPS-stimulated RAW264.7 cells [54], while anti-inflammatory properties of EO from taxonomically close related bergamot (*Citrus bergamia* Risso) have been demonstrated *in vivo* using the carrageenan-induced rat paw oedema test [25].

Additionally, we found that all CEOs showed radical scavenging activity against the two most commonly used non-biological radicals. Interestingly, we observed that radical scavenging activity increased with maturation when measured against DPPH•, while an opposite trend was observed in the case of ABTS•^+^. It has been previously reported that antioxidant capacity determined by in vitro assays can significantly differ [55]. The ABTS assay is based on the *in situ* generation of a blue/green radical (ABTS•^+^) that can be reduced by antioxidants by either single electron transfer (SET) or hydrogen transfer (HT) mechanisms, whereas the DPPH assay is based on the reduction of the purple DPPH• to 1,1-diphenyl-2-picryl hydrazine (DPPH-H) via HT mechanism. Moreover, it is worth noticing that CEOs are coloured (CEO1 and CEO2 are green, while CEO3 is yellow). Differences between in vitro antioxidant capacities determined by the two assays have been reported for highly pigmented samples [55]. Nevertheless, the combination of the results obtained from these two assays is considered of higher significance for a reliable evaluation of plant extract radical scavenging ability than the use of only one method [56]. Remarkably, we found that CEO2 showed high radical scavenging activity in both assays, indicating that CEO2 can act against both DPPH• and ABTS•^+^. In line with our findings, anti-inflammatory potential is often associated with free radical scavenging activity [14,51,52,57]. Reactive oxygen species (ROS) are crucial in the onset of the inflammation by activating transcription factors, such as the nuclear factor-kappa B (NF-κB), which induces the gene expression of inflammatory enzymes and cytokines [1]. 

The analysis of the volatile composition revealed that limonene, linalool, and linalyl acetate were the main components in all CEOs, while γ-terpinene was found in a significant percentage in CEO2 and to a lesser extent in CEO3. The relatively low amount of limonene compared to other *Citrus* species is in line with data from Lota and coworkers [32], while it is in contrast with other results, where limonene represented 80.1% of the oil [33]. Interestingly, linalool and linalyl acetate were reported to be absent or present in far lower amounts in previous works [32,33]. These variations could be due to differences in ripening stage, genetic origins, or pedoclimatic conditions. In line with the previously mentioned close taxonomical relation between chinotto and bergamot (*Citrus bergamia* Risso) [19,20], our results were comparable to the known distribution of volatile compounds in bergamot essential oil, comprising a relatively low amount of limonene (25–53%) and high percentages of linalool (2–20%), linalyl acetate (15–40%), and γ-terpinene [25]. Regarding individual components, limonene is by far the most abundant compound in CEO2 and CEO3 and these results can be associated with the corresponding radical scavenging activities, as limonene has been reported to exert strong DPPH• and poor ABTS•^+^ scavenging abilities [58]. Moreover, this monoterpene is the main bioactive molecule of many essential oils known to display anti-inflammatory properties [53,59,60]. However, it is not possible to ascribe the reduction of NO concentration to limonene only, since CEO3 contains amounts comparable to those of CEO2 (Table 1). Other compounds identified in CEO2, including linalool, linalyl acetate, and γ-terpinene, have been previously reported to display antioxidant and anti-inflammatory activities *in vitro* and in vivo [51,61,62,63,64]. In particular, linalool, and to a lesser extent linalyl acetate, have been found to reduce carrageenan-induced paw edema in rats [62] and to inhibit NO, IL-6 and TNF-α production and gene expression in LPS-stimulated RAW264.7 cells [63]. γ-Terpinene treatment reduced carrageenan-induced paw edema and neutrophil migration, as well as production of pro-inflammatory cytokines, such as interleukin (IL)-1β and tumor necrosis factor-α (TNF-α), in a carrageenan-induced peritonitis model and cell migration in LPS-induced lung injury in Swiss mice [64]. Moreover, γ-terpinene was found to reduce the production of pro-inflammatory cytokines, such as IL-1β and IL-6, and to enhance that of the anti-inflammatory cytokine, IL-10, in LPS-induced murine peritoneal macrophages [64]. However, its concentration in CEO2 is lower than that previously reported to reduce NO [51]. Therefore, a possible explanation is that the reduction of the NO levels elicited by CEO2 could originate from the additive and/or synergistic effects of more bioactives, rather than from a specific compound. The sum of limonene, linalool, linalyl acetate, and γ-terpinene accounted for 93.0% of the total constituents in CEO2. Such a high percentage suggests that these four compounds might be responsible for its observed anti-inflammatory activity. In line with these findings, synergistic effects of the components of other *Citrus* essential oils in murine macrophages have been previously reported [52,54]. Nevertheless, a variation in the individual amounts upon maturation was found and it is likely that this plays a role in the observed differences in bioactivity. In particular, γ-terpinene was found in a significant percentage only in CEO2 and, as such, could play a crucial role in the observed anti-inflammatory properties. 

In conclusion, in the present study, we show that, for the first time, similar to bergamot (*Citrus bergamia* Risso), extracts from chinotto (*Citrus myrtifolia* Raf.) also possess potent in vitro anti-inflammatory properties. In contrast to bergamot, which only grows in restricted areas due to its relatively poor ability to adapt, chinotto has been shown to easily adapt to different environmental conditions and, as such, might be of higher economical interest. Interestingly, the level of bioactivity was found to greatly depend on ripening stage of the fruit, since only the essential oil from half ripe fruits (CEO2) effectively reduced NO levels and gene-expression of the LPS-induced pro-inflammatory mediators, COX-2, iNOS, IL-1β, IL-6, and MCP-1 in RAW 264.7 macrophages. Moreover, CEO2 exhibited high radical scavenging activity against two commonly used non-biological radicals (DPPH• and ABTS•^+^). Limonene, linalool, linalyl acetate, and γ-terpinene were identified as the main components in CEO2 and our data suggest additive and/or synergistic effects of these compounds. Our findings warrant further studies to explore the potential of chinotto to improve human health and, when positive, to guide selection of most optimal mixtures. To this end, an important issue would be to study the oral bioavailability of the components. Only limited data are available, suggesting that limonene is absorbed from the GI tract, but data on linalool, linalyl acetate, and γ-terpinene appear to be lacking. In view of this, combination effects may also occur.

## Figures and Tables

**Table 1:** Volatile components identified in CEOs.

No.	Constituent	CAS nr	Peak Area (%) ^a^		
			CEO1	CEO2	CEO3
1	β-Pinene	127-91-3	<0.1 a	0.5 b	0.5 b
2	Limonene	5989-27-5	26.9 a	54.3 b	48.7 b
3	Sabinene	3387-41-5	0.2 a,b	0.1 a	0.3 b
4	Myrcene	123-35-3	0.1 a	0.6 b	1.2 c
5	γ-Terpinene	99-85-4	<0.1 a	3.7 b	0.4 c
7	Linalool	78-70-6	19.6 a	12.1 b	32.4 c
8	Neral	5392-40-5	0.2 a	0.3 a,b	0.5 b
9	Linalyl acetate	115-95-7	47.5 a	22.9 b	12.0 c
10	Geranial	5392-40-5	0.9 a	0.5 b	0.8 a
11	Geranyl acetate	105-87-3	1.0 a	0.3 b	0.2 b
12	β-Caryophyllene	87-44-5	0.3 a,b	0.4 b	0.1 c
Total identified			96.7	95.9	97.5

^a^ For each chinotto essential oil (CEO), data presented are the mean values of three independent experiments. Shared letters in the same rows show no statistical significance (*p* > 0.05), while different letters designate significant differences (*p* < 0.05).

**Table 2:** Radical scavenging activities of CEOs.

CEO	Ripening Stage	Scavenging Activity ^a^	
		DPPH•	ABTS•^+^
CEO1	Green	6.1 ± 0.8 a	11.1 ± 0.1 a
CEO2	Half ripe	7.8 ± 0.2 b	10.8 ± 0.3 a
CEO3	Ripe	8.1 ± 0.4 b	9.4 ± 0.1 b

^a^ µmol of Trolox Equivalents (TE)/g of CEO. Shared letters in the same columns show no statistical significance (*p* > 0.05), while different letters designate significant differences (*p* < 0.05).

## References

[1] Finkel T., Holbrook N.J. (2000). Oxidants, oxidative stress and the biology of ageing. Nature.

[2] Medzhitov R. (2008). Origin and physiological roles of inflammation. Nature.

[3] Nathan C. (2002). Points of control in inflammation. Nature.

[4] Serhan C.N., Savill J. (2005). Resolution of inflammation: The beginning programs the end. Nat. Immunol..

[5] Valledor A.F., Comalada M., Santamaria-Babi L.F., Lloberas J., Celada A. (2010). Macrophage proinflammatory activation and deactivation: A question of balance. Adv. Immunol..

[6] Li X., Lian L.H., Bai T., Wu Y.L., Wan Y., Xie W.X., Jin X., Nan J.X. (2011). Cryptotanshinone inhibits LPS-induced proinflammatory mediators via TLR4 and TAK1 signaling pathway. Int. Immunopharmacol..

[7] Li X., Xu W. (2011). TLR4-mediated activation of macrophages by the polysaccharide fraction from *Polyporus umbellatus*(pers) Fries. J. Ethnopharmacol..

[8] Barton G.M. (2008). A calculated response: Control of inflammation by the innate immune system. J. Clin. Investig..

[9] Bosca L., Zeini M., Traves P.G., Hortelano S. (2005). Nitric oxide and cell viability in inflammatory cells: A role for NO in macrophage function and fate. Toxicology.

[10] Sergent T., Piront N., Meurice J., Toussaint O., Schneider Y.J. (2010). Anti-inflammatory effects of dietary phenolic compounds in an in vitro model of inflamed human intestinal epithelium. Chem.-Biol. Int..

[11] Zorrilla P., Rodriguez-Nogales A., Algieri F., Garrido-Mesa N., Olivares M., Rondon D., Zarzuelo A., Utrilla M.P., Galvez J., Rodriguez-Cabezas M.E. (2014). Intestinal anti-inflammatory activity of the polyphenolic-enriched extract Amanda® in the trinitrobenzenesulphonic acid model of rat colitis. J. Funct. Foods.

[12] Bakkali F., Averbeck S., Averbeck D., Idaomar M. (2008). Biological effects of essential oils—A review. Food Chem. Toxicol..

[13] Sharifi-Rad J., Sureda A., Tenore G.C., Daglia M., Sharifi-Rad M., Valussi M., Tundis R., Sharifi-Rad M., Loizzo M.R., Ademiluyi A.O. (2017). Biological Activities of Essential Oils: From Plant Chemoecology to Traditional Healing Systems. Molecules.

[14] Chou S.-T., Lai C.-P., Lin C.-C., Shih Y. (2012). Study of the chemical composition, antioxidant activity and anti-inflammatory activity of essential oil from *Vetiveria zizanioides*. Food Chem..

[15] Jayasena D.D., Jo C. (2013). Essential oils as potential antimicrobial agents in meat and meat products: A review. Trends Food Sci. Technol..

[16] Dugo G., Di Giacomo A. (2002). Citrus—The Genus Citrus.

[17] Liu Y., Heying E., Tanumihardjo S.A. (2012). History, global distribution, and nutritional importance of citrus fruits. Compr. Rev. Food Sci. Food Saf..

[18] Barreca D., Bellocco E., Caristi C., Leuzzi U., Gattuso G. (2010). Flavonoid composition and antioxidant activity of juices from chinotto (*Citrus* x *myrtifolia* Raf.) fruits at different ripening stages. J. Agric. Food Chem..

[19] Gattuso G., Barreca D., Caristi C., Gargiulli C., Leuzzi U. (2007). Distribution of flavonoids and furocoumarins in juices from cultivars of *Citrus bergamia* Risso. J. Agric. Food Chem..

[20] Taverna D., Di Donna L., Mazzotti F., Tagarelli A., Napoli A., Furia E., Sindona G. (2016). Rapid discrimination of bergamot essential oil by paper spray mass spectrometry and chemometric analysis. J. Mass Spectrom..

[21] Borgatti M., Mancini I., Bianchi N., Guerrini A., Lampronti I., Rossi D., Sacchetti G., Gambari R. (2011). Bergamot (*Citrus bergamia* Risso) fruit extracts and identified components alter expression of interleukin 8 gene in cystic fibrosis bronchial epithelial cell lines. BMC Biochem..

[22] Ferlazzo N., Cirmi S., Calapai G., Ventura-Spagnolo E., Gangemi S., Navarra M. (2016). Anti-Inflammatory Activity of *Citrus bergamia* Derivatives: Where Do We Stand?. Molecules.

[23] Impellizzeri D., Cordaro M., Campolo M., Gugliandolo E., Esposito E., Benedetto F., Cuzzocrea S., Navarra M. (2016). Anti-inflammatory and antioxidant effects of flavonoid-rich fraction of bergamot juice (BJe) in a mouse model of intestinal ischemia/reperfusion injury. Front. Pharmacol..

[24] Impellizzeri D., Bruschetta G., Di Paola R., Ahmad A., Campolo M., Cuzzocrea S., Esposito E., Navarra M. (2015). The anti-inflammatory and antioxidant effects of bergamot juice extract (BJe) in an experimental model of inflammatory bowel disease. Clin. Nutr..

[25] Navarra M., Mannucci C., Delbò M., Calapai G. (2015). *Citrus bergamia* essential oil: From basic research to clinical application. Front. Pharmacol..

[26] Risitano R., Currò M., Cirmi S., Ferlazzo N., Campiglia P., Caccamo D., Ientile R., Navarra M. (2014). Flavonoid fraction of bergamot juice reduces LPS-induced inflammatory response through SIRT1-mediated NF-κB inhibition in THP-1 monocytes. PLoS ONE.

[27] Sommella E., Pepe G., Pagano F., Tenore G.C., Marzocco S., Manfra M., Calabrese G., Aquino R.P., Campiglia P. (2014). UHPLC profiling and effects on LPS-stimulated J774A.1 macrophages of flavonoids from bergamot (*Citrus bergamia*) juice, an underestimated waste product with high anti-inflammatory potential. J. Funct. Foods.

[28] Barreca D., Bellocco E., Caristi C., Leuzzi U., Gattuso G. (2011). Distribution of *C*- and *O*-glycosyl flavonoids, (3-hydroxy-3-methylglutaryl)glycosyl flavanones and furocoumarins in *Citrus aurantium* L. Juice. Food Chem..

[29] Barreca D., Bellocco E., Caristi C., Leuzzi U., Gattuso G. (2011). Elucidation of the flavonoid and furocoumarin composition and radical-scavenging activity of green and ripe chinotto (*Citrus myrtifolia* Raf.) fruit tissues, leaves and seeds. Food Chem..

[30] Protti M., Valle F., Poli F., Raggi M.A., Mercolini L. (2015). Bioactive molecules as authenticity markers of Italian Chinotto (*Citrus × myrtifolia*) fruits and beverages. J. Pharm. Biomed. Anal..

[31] Scordino M., Sabatino L., Belligno A., Gagliano G. (2011). Preliminary Study on Bioactive Compounds of *Citrus × myrtifolia Rafinesque* (Chinotto) to Its Potential Application in Food Industry. Food Nutr. Sci..

[32] Lota M.-L., de Rocca Serra D., Jacquemond C., Tomi F., Casanova J. (2001). Chemical variability of peel and leaf essential oils of sour orange. Flavour Fragr. J..

[33] Chialva F., Doglia G. (1990). Essential Oil Constituents of Chinotto (*Citrus aurantium* L. var. myrtifolia Guill.). J. Essent. Oil Res..

[34] Gabriele B., Fazio A., Dugo P., Costa R., Mondello L. (2009). Essential oil composition of *Citrus medica* L. Cv. Diamante (*Diamante citron*) determined after using different extraction methods. J. Sep. Sci..

[35] Fazio A., Caroleo M.C., Cione E., Plastina P. (2017). Novel acrylic polymers for food packaging: Synthesis and antioxidant properties. Food Packag. Shelf Life.

[36] Re R., Pellegrini N., Proteggente A., Pannala A., Yang M., Rice-Evans C. (1996). Antioxidant activity applying an improved ABTS radical cation decolorization assay. Free Radic. Biol. Med..

[37] Wang Y., Plastina P., Vincken J.-P., Jansen R., Balvers M., ten Klooster J.P., Gruppen H., Witkamp R., Meijerink J. (2017). *N*-Docosahexaenoyl Dopamine, an Endocannabinoid-like Conjugate of Dopamine and the n-3 Fatty Acid Docosahexaenoic Acid, Attenuates Lipopolysaccharide-Induced Activation of Microglia and Macrophages via COX-2. ACS Chem. Neurosci..

[38] Bogdan C. (2001). Nitric oxide and the immune response. Nat. Immunol..

[39] Moncada S., Higgs A. (1993). The l-arginine-nitric oxide pathway. N. Eng. J. Med..

[40] Meijerink J., Poland M., Balvers M.G.J., Plastina P., Lute C., Dwarkasing J., van Norren K., Witkamp R.F. (2015). Inhibition of COX-2-mediated eicosanoid production plays a major role in the anti-inflammatory effects of the endocannabinoid *N*-docosahexaenoylethanolamine (DHEA) in macrophages. Br. J. Pharmacol..

[41] Chang Y.C., Li P.C., Chen B.C., Chang M.S., Wang J.L., Chiu W.T., Lin C.H. (2006). Lipoteichoic acid-induced nitric oxide synthase expression in RAW 264.7 macrophages is mediated by cyclooxygenase-2, prostaglandin E2, protein kinase A, p38 MAPK, and nuclear factor-kappaB pathways. Cell Signal..

[42] Cerella C., Sobolewski C., Dicato M., Diederich M. (2010). Targeting COX-2 expression by natural compounds: A promising alternative strategy to synthetic COX-2 inhibitors for cancer chemoprevention and therapy. Biochem. Pharmacol..

[43] Furia E., Napoli A., Tagarelli A., Sindona G. (2013). Speciation of 2-hydroxybenzoic acid with calcium(II), magnesium(II), and nickel(II) cations in self-medium. J. Chem. Eng. Data.

[44] Kim E.Y., Moudgil K.D. (2008). Regulation of autoimmune inflammation by pro-inflammatory cytokines. Immunol. Lett..

[45] Laflamme N., Lacroix S., Rivest S. (1999). An Essential Role of Interleukin-1β in Mediating NF-kB Activity and COX-2 Transcription in Cells of the Blood–Brain Barrier in Response to a Systemic and Localized Inflammation But Not During Endotoxemia. J. Neurosci..

[46] Ren K., Torres R. (2009). Role of interleukin-1beta during pain and inflammation. Brain Res. Rev..

[47] Lupinacci E., Meijerink J., Vincken J.P., Gabriele B., Gruppen H., Witkamp R.F. (2009). Xanthohumol from Hop (*Humulus lupulus* L.) Is an Efficient Inhibitor of Monocyte Chemoattractant Protein-1 and Tumor Necrosis Factor-alpha Release in LPS-Stimulated RAW 264.7 Mouse Macrophages and U937 Human Monocytes. J. Agric. Food Chem..

[48] Burkovská A., Čikoš Š., Juhás Š., Il’Ková G., Rehák P., Koppel J. (2007). Effects of a combination of thyme and oregano essential oils on TNBS-induced colitis in mice. Mediat. Inflamm..

[49] Hotta M., Nakata R., Katsukawa M., Hori K., Takahashi S., Inoue H. (2010). Carvacrol, a component of thyme oil, activates PPARα and γ and suppresses COX-2 expression. J. Lipid Res..

[50] Yoon W.J., Moon J.Y., Song G., Lee Y.K., Han M.S., Lee J.S., Ihm B.S., Lee W.J., Lee N.H., Hyun C.G. (2010). *Artemisia fukudo* essential oil attenuates LPS-induced inflammation by suppressing NF-κB and MAPK activation in RAW264.7 macrophages. Food Chem. Toxicol..

[51] Kim K.-N., Ko Y.-J., Yang H.-M., Ham Y.-M., Roh S.W., Jeon Y.-J., Ahn G., Kang M.-C., Yoon W.-J., Kim D. (2013). Anti-inflammatory effect of essential oil and its constituents from fingered citron (*Citrus medica* L. var. *sarcodactylis*) through blocking JNK, ERK and NF-kB signaling pathways in LPS-activated RAW 264.7 cells. Food Chem. Toxicol..

[52] Miguel M.G. (2010). Antioxidant and Anti-Inflammatory Activities of Essential Oils: A Short Review. Molecules.

[53] Amorim J.L., Simas D.L.R., Pinheiro M.M.G., Moreno D.S.A., Alviano C.S., da Silva A.J.R., Dias Fernandes P. (2016). Anti-Inflammatory Properties and Chemical Characterization of the Essential Oils of Four Citrus Species. PLoS ONE.

[54] Shen C.-Y., Jiang J.-G., Zhu W., Ou-Yang Q. (2017). Anti-inflammatory Effect of Essential Oil from *Citrus aurantium* L. var. *amara* Engl. J. Agric. Food Chem..

[55] Floegel A., Kim D.-O., Chung S.-J., Koo S.I., Chun O.K. (2011). Comparison of ABTS/DPPH assays to measure antioxidant capacity in popular antioxidant-rich US foods. J. Food Compos. Anal..

[56] Surveswaran S., Cai Y.-Z., Corke H., Sun M. (2007). Systematic evaluation of natural phenolic antioxidants from 133 Indian medicinal plants. Food Chem..

[57] Fazio A., Plastina P., Meijerink J., Witkamp R.F., Gabriele B. (2013). Comparative analyses of seeds of wild fruits of Rubus and Sambucus species from Southern Italy: Fatty acid composition of the oil, total phenolic content, antioxidant and anti-inflammatory properties of the methanolic extracts. Food Chem..

[58] Yang S.-A., Jeon S.-K., Lee E.-J., Im N.-K., Jhee K.-H., Lee S.-P., Lee I.-S. (2009). Radical Scavenging Activity of the Essential Oil of Silver Fir (*Abies alba*). J. Clin. Biochem. Nutr..

[59] Ku C.-M., Lin J.-Y. (2013). Anti-inflammatory effects of 27 selected terpenoid compounds tested through modulating Th1/Th2 cytokine secretion profiles using murine primary splenocytes. Food Chem..

[60] Miller J.A., Thompson P.A., Hakim I.A., Chow H.H.S., Thomson C.A. (2011). d-Limonene: A bioactive food component from citrus and evidence for a potential role in breast cancer prevention and treatment. Oncol. Rev..

[61] Yang S.-A., Jeon S.-K., Lee E.-J., Shim C.-H., Lee I.-S. (2010). Comparative study of the chemical composition and antioxidant activity of six essential oils and their components. Nat. Prod. Res..

[62] Peana A.T., D’Aquila P.S., Panin F., Serra G., Pippia P., Moretti M.D.L. (2002). Anti-inflammatory activity of linalool and linalyl acetate constituents of essential oils. Phytomedicine.

[63] Maeda H., Yamazaki M., Katagata Y. (2013). Kuromoji (*Lindera umbellata*) Essential Oil Inhibits LPS-Induced Inflammation in RAW 264.7 Cells. Biosci. Biotechnol. Biochem..

[64] De Oliveira Ramalho T.R., Filgueiras L.R., de Oliveira M.T.P., de Araujo Lima A.L., Bezerra-Santos C.R., Jancar S., Piuvezam M.R. (2016). Gamma-Terpinene Modulation of LPS-Stimulated Macrophages is Dependent on the PGE2/IL-10 Axis. Planta Med..

